# Supplementary material for: Causal Relationship between Aging and Anorexia Nervosa: A White-Matter-Microstructure-Mediated Mendelian Randomization Analysis
Source: Biomedicines. 2024 Aug 16;12(8):1874. doi: 10.3390/biomedicines12081874 (PMC11351342; doi:10.3390/biomedicines12081874)
Supplement: Supplementary file 1 [file biomedicines-12-01874-s001.zip › Supplementary Figure/Figure S4/Singe SNP plot of MR analysis for TL to ubm-b-1598.pdf]

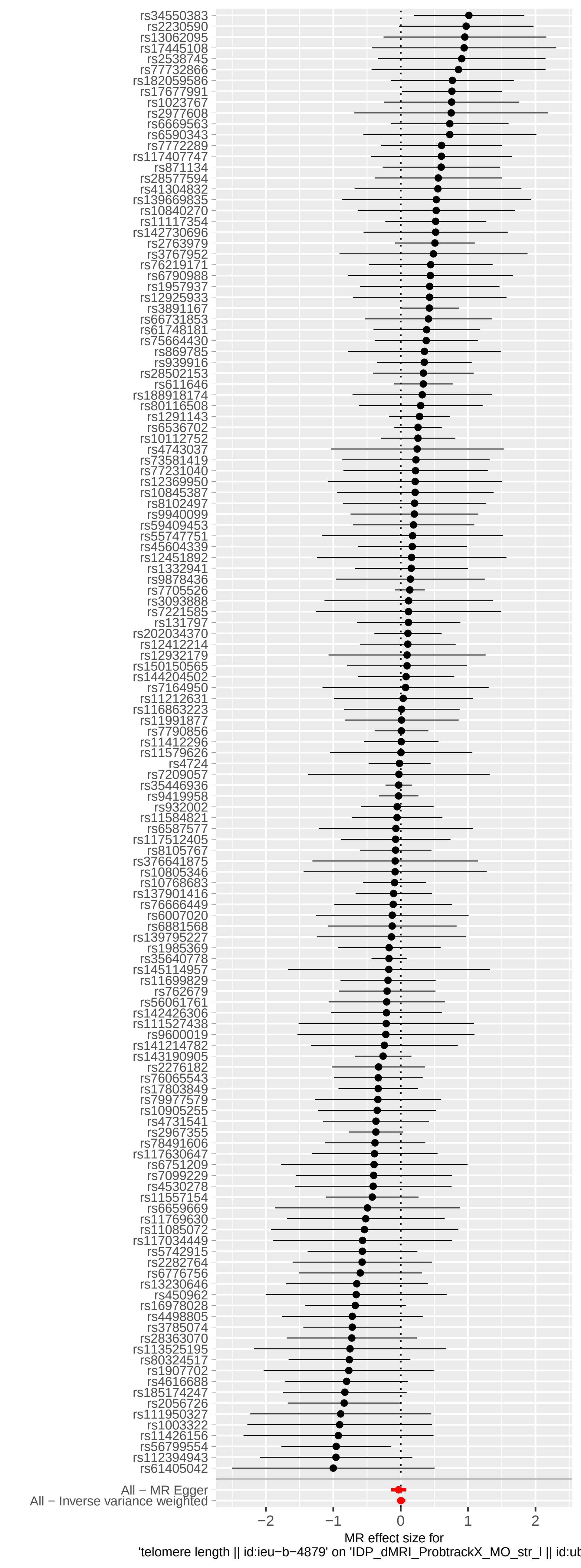
rs34550383
rs2230590
rs13062095
rs17445108
rs2538745
rs77732866
rs182059586
rs17677991
rs1023767
rs2977608
rs6669563
rs6590343
rs7772289
rs117407747
rs871134
rs28577594
rs41304832
rs139669835
rs10840270
rs11117354
rs142730696
rs2763979
rs3767952
rs76219171
rs6790988
rs1957937
rs12925933
rs3891167
rs66731853
rs61748181
rs75664430
rs869785
rs939916
rs28502153
rs611646
rs188918174
rs80116508
rs1291143
rs6536702
rs10112752
rs4743037
rs73581419
rs77231040
rs12369950
rs10845387
rs8102497
rs9940099
rs59409453
rs55747751
rs45604339
rs12451892
rs1332941
rs9878436
rs7705526
rs3093888
rs7221585
rs131797
rs202034370
rs12412214
rs12932179
rs150150565
rs144204502
rs7164950
rs11212631
rs116863223
rs11991877
rs7790856
rs11412296
rs11579626
rs4724
rs7209057
rs35446936
rs9419958
rs932002
rs11584821
rs6587577
rs117512405
rs8105767
rs376641875
rs10805346
rs10768683
rs137901416
rs76666449
rs6007020
rs6881568
rs139795227
rs1985369
rs35640778
rs145114957
rs11699829
rs762679
rs56061761
rs142426306
rs111527438
rs9600019
rs141214782
rs143190905
rs2276182
rs76065543
rs17803849
rs79977579
rs10905255
rs4731541
rs2967355
rs78491606
rs117630647
rs6751209
rs7099229
rs4530278
rs11557154
rs6659669
rs11769630
rs11085072
rs117034449
rs5742915
rs2282764
rs6776756
rs13230646
rs450962
rs16978028
rs4498805
rs3785074
rs28363070
rs113525195
rs80324517
rs1907702
rs4616688
rs185174247
rs2056726
rs111950327
rs1003322
rs11426156
rs56799554
rs112394943
rs61405042
All − MR Egger
All − Inverse variance weighted
−2
−1
0
1
2
MR effect size for
'telomere length || id:ieu−b−4879' on 'IDP_dMRI_ProbtrackX_MO_str_l || id:ub
